# Readability of the American, Canadian, and British Otolaryngology–Head and Neck Surgery Societies’ Patient Materials

**DOI:** 10.1177/01945998211033254

**Published:** 2021-08-10

**Authors:** Joo Hyun Kim, Elysia Grose, Justine Philteos, David Forner, Christopher W. Noel, Vincent Wu, Antoine Eskander

**Affiliations:** 1 Faculty of Medicine, University of Toronto, Toronto, Ontario, Canada; 2 Department of Otolaryngology–Head & Neck Surgery, University of Toronto, Toronto, Ontario, Canada; 3 Department of Otolaryngology–Head & Neck Surgery, Dalhousie University, Halifax, Nova Scotia, Canada; 4 Institute of Health Policy, Management and Evaluation, Dalla Lana School of Public Health, University of Toronto, Toronto, Ontario, Canada

**Keywords:** readability, patient education, health literacy, otolaryngology

## Abstract

**Objective:**

Patient education materials across 3 national English otolaryngology–head and neck surgery (OHNS) societies: the American Academy of Otolaryngology–Head and Neck Surgery (AAO-HNS), the Canadian Society of Otolaryngology–Head and Neck Surgery (CSOHNS), and Ear, Nose, and Throat United Kingdom (ENT UK) were examined to determine whether they are written at a level suitable for patient comprehension.

**Study Design:**

Cross-sectional study.

**Setting:**

Online patient materials presented through OHNS national societies.

**Methods:**

Readability was calculated using the Flesch-Kincaid Grade Level, Flesch-Kincaid Reading Ease Score, and Simple Measure of Gobbledygook Index. All public patient education materials available through the CSOHNS, AAO-HNS, and ENT UK websites were assessed. Patient education materials were grouped into categories by subspecialty.

**Results:**

In total, 128 patient materials from the 3 societies were included in the study. All 3 societies required a minimum grade 9 reading comprehension level to understand their online materials. According to Flesch-Kincaid Grade Level, the CSOHNS required a significantly higher reading grade level to comprehend the materials presented when compared to AAO-HNS (11.3 vs 9.9; 95% CI, 0.5-2.4; *P* < .01) and ENT UK (11.3 vs 9.4; 95% CI, 0.9-2.9; *P* < .01). Patient education materials related to rhinology were the least readable among all 3 societies.

**Conclusion:**

This study suggests that the reading level of the current patient materials presented through 3 national OHNS societies are written at a level that exceeds current recommendations. Promisingly, it highlights an improvement for the readability of patient materials presented through the AAO-HNS.

One in 5 patients obtain medical information about their disease or surgical procedure from the Internet prior to their appointment with an otolaryngologist–head and neck surgeon.^1-3^ It is estimated that approximately 54% of patients with head and neck cancer use the Internet to find information about their treatments.^4-6^ A single-institutional study exploring Internet use among otolaryngology–head and neck surgery (OHNS) patients concluded that approximately 61% of patients conducted an Internet search with regards to their condition before seeing a general otolaryngologist, 60% before a rhinologist, 44% before seeing a laryngologist, and 52% before seeing a neurotologist.^2^ With patients becoming increasingly dependent on web-based health information,^7,8^ having online educational materials that can be understood by the patient population is of the utmost importance.

Health literacy, the degree to which an individual has the ability to understand and apply basic health information, has been established as one of the strongest predictors of overall health.^9-12^ Low health literacy is associated with poor health outcomes, including increased mortality rates and hospitalizations.^13-15^ This is of great concern since as high as 49% of Canadians,^16,17^ 36% of Americans,^18,19^ and 59% of Europeans^20,21^ have low literacy and limited ability to understand the health information presented to them. Within OHNS, many studies have demonstrated that available online information has been deficient in readability and is not written at a level easily understood by the public.^22-24^ However, some of these materials were not necessarily intended for patient education specifically, and thus commonly used readability targets may have been unnecessary.

The American Medical Association (AMA) has recommended that medical information written for patients should aim for a reading level less than or equal to sixth grade as the average American reads at approximately an eighth-grade reading level.^25^ Similarly, in Canada and the United Kingdom, it is estimated that 48% of Canadians have literacy levels that fall below a high school level, and 16% of adults in Northern Ireland and England read below the lowest level of reading proficiency, which equates to the estimated reading level of an individual 5 to 7 years of age.^26,27^ Previous studies have shown that OHNS web-based health information does not fulfill AMA recommendations.^28-30^ The Canadian Society of Otolaryngology–Head and Neck Surgery (CSOHNS), the American Academy of Otolaryngology–Head and Neck Surgery (AAO-HNS), and Ear, Nose, and Throat United Kingdom (ENT UK) have materials specifically targeted for patient education. However, the readability of CSOHNS and ENT UK materials has yet to be established, and the readability of the AAO-HNS was last assessed in 2016.^31^ Herein, we aimed to analyze the readability of the patient resources made available through 3 of the largest English-speaking national societies, as physicians often turn their patients to these resources for further information.

## Methods

All public patient education materials available through the CSOHNS, AAO-HNS, and ENT UK websites were assessed. Patient education materials were grouped into categories by subspecialty: otology, rhinology, head and neck oncologic surgery, and other. The other category included topics related to general OHNS, facial plastics, laryngology, and pediatric OHNS given the low number of patient education material in each individual category (see Supplementary Material 1 in the online version of the article). This study was exempted from the University of Toronto review board review because the educational material on the website is publicly accessible and does not involve patient records.

### Readability Evaluation

The text from each webpage was pasted to Microsoft Word (Microsoft Corp) and removed of any formatting elements that might interfere with readability assessment (headings, symbols, author information, references, etc). The plain text from each webpage was then assessed using an online readability calculator (https://readable.com/), as demonstrated in other readability studies.^29,31,32^ This resource evaluates the text’s readability based on criteria such as the average number of syllables and sentence length and yields the following readability scores: Flesch-Kincaid Grade Level (FKGL), the Flesch-Kincaid Reading Ease (FRE) score, and Simple Measure of Gobbledygook Index (SMOG) reading grade. The formulas can be found in Supplementary Material 2 (in the online version of the article). The FKGL is the most validated readability formula used for analyzing medical texts, and both the FRE and FKGL have been widely used to assess readability in the OHNS literature.^22,23,33-36^ FKGL approximates the reading grade level necessary to comprehend the text (eg, a score of 6 corresponds to a sixth-grade reading level). The FRE determines the readability using a score from 0 to 100, with higher scores indicating a higher level of readability, as shown in **Table 1**. The SMOG reading grade is a measure of readability that estimates the years of education needed to understand a piece of writing.^37^ Materials with low FKGL, high FRE, and low SMOG scores are considered more readable.

**Table 1.** Flesch-Kincaid Reading Ease (FRE) Readability Score and Interpretation.

FRE score	Interpretation
90-100	Very easy
80-89	Easy
70-79	Fairly easy
60-69	Standard
50-59	Fairly difficult
30-49	Difficult
<30	Very confusing

### Statistical Analyses

Categorical variables were reported using frequencies and proportions. Continuous variables were presented as mean and SD. Normalcy was assessed through the Shapiro-Wilk test and visual inspection of histogram and quantile-quantile plots. Comparison of continuous variables was performed using 1-way analysis of variance (ANOVA) with post hoc Tukey test. Comparisons of readability scores were made between CSOHNS, AAO-HNS, and ENT UK, along with different OHNS subspecialties (otology, rhinology, head and neck oncology, and other). A threshold of *P* < .05 was considered statistically significant. The effect sizes were reported using standardized mean differences (Cohen’s *d*).^38^ The absolute value of the standardized mean differences was categorized as small (0.2-0.5), medium (0.5-0.8), or large (≥0.8).^39^ Statistical analysis was performed using SPSS (v.26.0; SPSS, Inc).

## Results

In total, 128 online patient materials from 3 different national societies were included in the study: 63 from the AAO-HNS, 20 from the CSOHNS, and 45 from ENT UK.

The mean FRE, FKGL, and SMOG scores across the 3 societies are outlined in **Table 2**. The FRE scores corresponded to a reading interpretation of “fairly difficult” for ENT UK and AAO-HNS, whereas the FRE score for CSOHNS demonstrated a “difficult” interpretation. Furthermore, ENT UK had a significantly higher FRE score, compared to AAO-HNS (58.1 vs 50.6; *d =* 0.8, *P* < .01) and CSOHNS (58.1 vs 45.2; *d* = 1.3, *P* < .01). With regards to the SMOG analysis, CSOHNS was the least readable with a mean SMOG score corresponding to an undergraduate reading comprehension level (mean [SD], 13.5 [1.5]). ENT UK was the most readable according to the SMOG score and yet required a high school junior literacy on average (mean [SD], 11.8 [1.5]).

**Table 2.** FRE, FKGL, and SMOG scores for American Academy of Otolaryngology–Head and Neck Surgery, Canadian Society of Otolaryngology–Head and Neck Surgery, and Ear, Nose, and Throat United Kingdom.

Society	FRE, mean (SD)	FKGL, mean (SD)	SMOG, mean (SD)
American Academy of Otolaryngology–Head and Neck Surgery (n = 63)	50.6 (8.0)	9.9 (1.3)	12.2 (1.1)
Canadian Society of Otolaryngology–Head and Neck Surgery (n = 20)	45.2 (10.2)	11.3 (1.8)	13.5 (1.5)
Ear, Nose, and Throat United Kingdom (n = 45)	58.1 (9.9)	9.4 (1.8)	11.8 (1.5)

Abbreviations: FKGL, Flesch-Kincaid Grade Level; FRE, Flesch-Kincaid Reading Ease; SMOG, Simple Measure of Gobbledygook Index.

**Figure 1.**
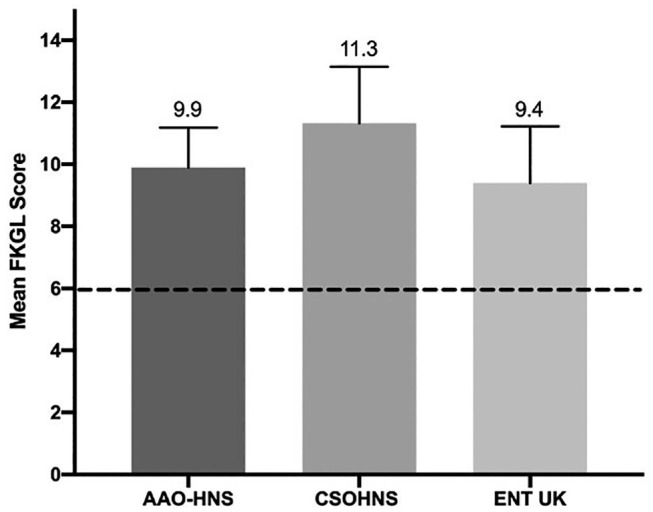
Mean FKGL scores for American Academy of Otolaryngology–Head and Neck Surgery (AAO-HNS), Canadian Society of Otolaryngology–Head and Neck Surgery (CSOHNS), and Ear, Nose, and Throat United Kingdom (ENT UK). The dotted line signifies the recommended sixth-grade reading level.

The FKGL grade-reading levels are outlined in **Figure 1**. According to FKGL scores, the CSOHNS required a significantly higher reading grade level to comprehend the materials presented when compared to AAO-HNS (11.3 vs 9.9; *d =* 1.00, *P* < .01) and ENT UK (11.3 vs 9.4; *d* = 1.3, *P* < .01). All 3 societies required a minimum grade 9 reading comprehension level to understand their online materials, as evidenced by their FKGL scores.

**Figure 2.**
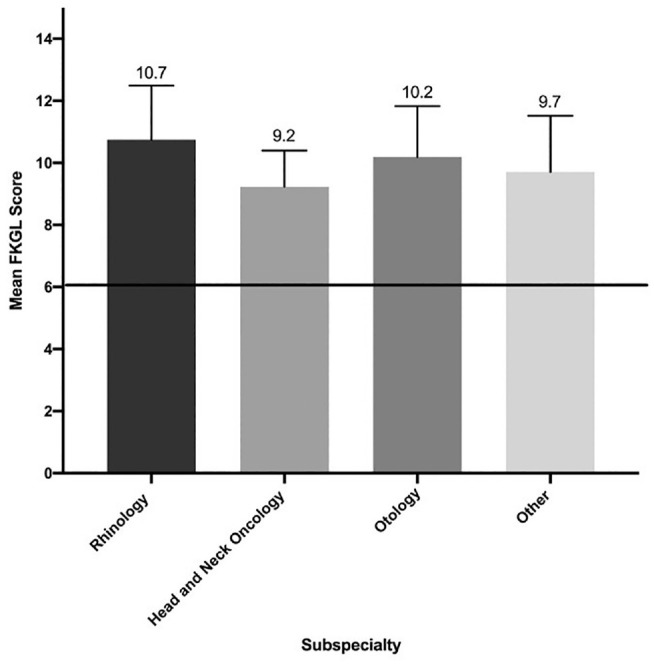
Mean Flesch-Kincaid Grade Level (FKGL) score per subspecialty across the societies. The solid line signifies the recommended sixth-grade reading level.

The mean FRE, FKGL, and SMOG scores for the OHNS subspecialties presented through the 3 societies is shown in **Table 3**. The patient materials on topics related to rhinology were the least readable between all 3 societies, as demonstrated in **Figure 2**. The rhinology patient materials from the CSOHNS had higher FKGL scores and lower FRE scores when compared with all other subspecialties and societies. The materials that were the most readable from the CSOHNS and AAO-HNS were in head and neck oncology with mean (SD) FRE scores of 57.5 (10.4) and 52.6 (7.2), respectively. Similarly, across the 3 societies (AAO-HNS, CSOHNS, and ENTUK, respectively), patient materials for head and neck oncology were the most readable according to SMOG analysis—mean (SD) of 11.8 (0.9), 12.1 (1.3), and 11.6 (1.1). The least readable subspecialty material corresponded to the rhinology materials presented through CSOHNS with a mean (SD) SMOG score of 14.3 (0.9).

**Table 3.** FRE, FKGL, and SMOG Scores per Subspecialty.

Society	Subspecialty	FRE, mean (SD)	FKGL, mean (SD)	SMOG, mean (SD)
American Academy of Otolaryngology–Head and Neck Surgery				
Otology (n = 16)		50.1 (8.1)	10.1 (1.2)	12.3 (1.1)
Rhinology (n = 10)		43.9 (6.5)	11.0 (0.9)	13.2 (0.8)
Head and neck oncology (n = 15)		52.6 (7.2)	9.4 (1.1)	11.8 (0.9)
Other (n = 22)		52.5 (7.9)	9.6 (1.3)	12.0 (1.1)
Canadian Society of Otolaryngology–Head and Neck Surgery				
Otology (n = 3)		41.8 (7.0)	11.7 (1.3)	13.4 (0.9)
Rhinology (n = 4)		41.5 (11.8)	12.4 (2.2)	14.3 (1.8)
Head and neck oncology (n = 3)		57.5 (10.4)	9.2 (1.4)	12.1 (1.3)
Other (n = 10)		44.0 (8.8)	11.4 (1.6)	13.7 (1.4)
Ear, Nose, and Throat United Kingdom				
Otology (n = 16)		55.2 (9.6)	10.0 (2.0)	12.3 (1.6)
Rhinology (n = 11)		55.2 (10.1)	10.0 (1.8)	12.0 (1.6)
Head and neck oncology (n = 9)		59.6 (7.5)	8.9 (1.3)	11.6 (1.1)
Other (n = 9)		65.1 (9.7)	8.1 (1.4)	10.9 (1.2)

Abbreviations: FKGL, Flesch-Kincaid Grade Level; FRE, Flesch-Kincaid Reading Ease; SMOG, Simple Measure of Gobbledygook Index.

## Discussion

In this study, it was demonstrated that the reading level of the current online patient materials presented through the 3 national OHNS societies was written at a level that exceeded current readability recommendations. The patient materials on topics related to rhinology were the least readable between all 3 societies.

Patient education is an integral component of patient-centered care and informed consent. Studies have shown that the use of online health information by patients can have a significant impact on the health care decision-making process.^5,13,40,41^ A study that set out to estimate the prevalence of poor reading skills in the OHNS outpatient population in the United Kingdom found that 28% of their patients had poor reading skills and were unable to comprehend medical leaflets.^42^ Moreover, studies on the utilization of Internet videos and websites for OHNS procedures have concluded they are of poor quality and difficult for patients to comprehend, making national society websites the ideal source for trusted patient education material.^43-46^ This requires OHNS societies to provide accurate information, written at a comprehensible level, as these are viewed as trusted sources of information for patient education.^29^

Our study concluded that neither AAO-HNS, CSOHNS, nor ENT UK had online patient materials written at a sixth-grade reading level as recommended by the AMA. Our current study has demonstrated that for each of these societies, across every subspecialty, the required reading grade level required is at a minimum of grade 11. Previous literature examining the materials presented through the AAO-HNS concluded that the online materials were written at a FKGL of 10.5 to 11.4.^31^ This ignited change as it is well established in the literature that patient comprehension of medical information is integral to their involvement in treatment decisions and improves patient outcomes.^47,48^ Our study demonstrated improvement in the FKGL reading levels of the material of the AAO-HNS, as the FKGL reading level was 9.8—this is encouraging as it demonstrates a decrease in reading difficulty of 1 to 2 academic grades. Furthermore, the online materials presented through CSOHNS and ENT UK have not been previously analyzed for their degree of readability. This study found that both patient materials from ENT UK and CSOHNS were found to have significantly higher FRE, FKGL, and SMOG scores, respectively, when compared to AAO-HNS. These findings highlight that there still remains room for improvement to make educational material on national society websites more comprehensible for patients.

With regards to our analysis of OHNS subspecialties, rhinology was unanimously the least readable topic across the 3 societies with reading grade levels ranging from the 10th grade to the 12th grade. These findings are in keeping with previous studies analyzing the readability of patient materials presented through the American Rhinology Society.^49^ One possible explanation for this is the inherent complexity embedded in rhinology, as concepts surrounding the location and conceptualization of the sinuses may be difficult for the average reader. Kasabwala et al^29^ analyzed patient materials from the AAO-HNS in 2012 and found that materials related to conditions of the nose and mouth had the highest percentage of complex words when compared to other categories. Wong and Levi^31^ in 2016 displayed methods of mitigating the medical jargon intrinsic to rhinology, including explaining the location and purposes of the sinuses before describing the symptoms of sinusitis.

It is estimated that 54% of patients with head and neck cancer use the Internet as a source of health information.^4^ This study also found that patient materials related to head and neck oncology from the AAO-HNS and CSOHNS were the most readable at approximately a ninth-grade reading level. This demonstrates an improvement from the study done by Kasabwala et al^29^ in 2012 as patient materials related to cancer from the AAO-HNS had an average FKGL score of 11.5. Although this is reassuring, studies have demonstrated that 12% to 47% of head and neck cancer survivors have inadequate health literacy^41,48,50^; as such, there remains room for improvement as many of these resources are written above the recommended sixth-grade reading level. A study analyzing the readability of online medical information looked at the online patient materials presented through the national societies for the numerous surgical subspecialties, and they compared the readability results to those presented through the AAO-HNS. They concluded that, similar to the AAO-HNS, the readability scores for the patient education materials from the various other surgical subspecialty websites were above the recommended sixth-grade level.^32^ Many patients access online patient education materials preconsultation without having prior discussions with health care professionals to provide a foundational understanding of their condition^51^; thus, patients should be encouraged and be given opportunities to bring in the materials that they have read independently for the physician to address any questions that have arisen from these resources.

As evidenced by the reading difficulty of the analyzed texts, writing production for conditions within OHNS is a difficult task. The results of this study highlight the need for improving the readability and the overall comprehension of patient education materials available on the websites of OHNS national societies. First, individuals with expertise in health literacy and patient education, such as specialized librarians, could be incorporated into the writing process to ensure readability.^52,53^ Patient engagement in the development of this material is critical, but many of the patients chosen for this task may have a higher level of education and may not reflect the target population.^54^ As such, patients with a wide range of educational backgrounds should be engaged in the production process. In addition, materials should be put through a readability software prior to publication to objectively evaluate their reading difficulty. Some other suggestions for improving the readability of patient education materials include limiting the number of words in sentences to a maximum of 8 to 10 words,^55^ using 1- to 2-syllable words,^56^ and replacing medical jargon with simple terms where possible.^57,58^ Supplemental Material 3 (in the online version of the article) provides examples of these additional suggestions implemented to further improve the readability of current text found on the 3 OHNS societies analyzed. In addition, some ways authors can improve readability that are not necessarily captured with readability scores include (1) writing in an active voice^59,60^; (2) the strategic use of formatting, including highlighting or bolding important points^61^; and (3) incorporating mixed media such as videos and images to improve the understanding of medical concepts.^62^ These findings further necessitate the need to employ strategies that ensure that the patient information available online is easily understood by the patient population at large.

There are a few limitations to our study. It is difficult to determine whether the patient education materials included in this study were intended for patients to read in the absence of discussions with their health care provider. FRE and FKGL are readability formulas that calculate the number of syllables and words in a sentence, and these may not adequately account for nuanced complexity of the text, which could potentially bias our results toward a conservative or lower readability score. Furthermore, readability is only 1 aspect of the appropriateness of written information. Availability, format, style, and content are all important factors that, if not addressed, can act as barriers to patient access. In addition, these assessment tools do not take into account videos, diagrams, and other presentation elements that contribute to the quality and understandability of the online patient materials.

## Conclusion

The findings of this study suggest that the reading level of the current online patient materials presented through 3 national OHNS societies (AAO-HNS, CSOHNS, and ENT UK) does not meet readability standards. Encouragingly, it highlights an improvement for the readability of patient materials presented through the AAO-HNS. Societies developing patient material should ensure that a professional writer and editor are included as part of the team to ensure readability as a key requirement. Ensuring resources are easily comprehensible has the potential to improve shared decision-making, patient satisfaction, and postoperative outcomes, all of which are important facets of patient-centered health care delivery. Thus, we hope the findings of this current study ignite revisions to the current patient materials presented through the AAO-HNS, CSOHNS, and ENT UK.

## Supplemental Material

sj-docx-1-oto-10.1177_01945998211033254 – Supplemental material for Readability of the American, Canadian, and British Otolaryngology–Head and Neck Surgery Societies’ Patient Materials Click here for additional data file. Supplemental material, sj-docx-1-oto-10.1177_01945998211033254 for Readability of the American, Canadian, and British Otolaryngology–Head and Neck Surgery Societies’ Patient Materials by Joo Hyun Kim, Elysia Grose, Justine Philteos, David Forner, Christopher W. Noel, Vincent Wu and Antoine Eskander in Otolaryngology–Head and Neck Surgery

sj-docx-2-oto-10.1177_01945998211033254 – Supplemental material for Readability of the American, Canadian, and British Otolaryngology–Head and Neck Surgery Societies’ Patient Materials Click here for additional data file. Supplemental material, sj-docx-2-oto-10.1177_01945998211033254 for Readability of the American, Canadian, and British Otolaryngology–Head and Neck Surgery Societies’ Patient Materials by Joo Hyun Kim, Elysia Grose, Justine Philteos, David Forner, Christopher W. Noel, Vincent Wu and Antoine Eskander in Otolaryngology–Head and Neck Surgery

sj-docx-3-oto-10.1177_01945998211033254 – Supplemental material for Readability of the American, Canadian, and British Otolaryngology–Head and Neck Surgery Societies’ Patient Materials Click here for additional data file. Supplemental material, sj-docx-3-oto-10.1177_01945998211033254 for Readability of the American, Canadian, and British Otolaryngology–Head and Neck Surgery Societies’ Patient Materials by Joo Hyun Kim, Elysia Grose, Justine Philteos, David Forner, Christopher W. Noel, Vincent Wu and Antoine Eskander in Otolaryngology–Head and Neck Surgery
