# Supplementary material for: Readability of the American, Canadian, and British Otolaryngology–Head and Neck Surgery Societies’ Patient Materials
Source: Otolaryngol Head Neck Surg. 2021 Aug 10;166(5):862–8. doi: 10.1177/01945998211033254 (PMC9066686; doi:10.1177/01945998211033254)
Supplement: sj-docx-1-oto-10.1177_01945998211033254 – Supplemental material for Readability of the American, Canadian, and British Otolaryngology–Head and Neck Surgery Societies’ Patient Materials [file sj-docx-1-oto-10.1177_01945998211033254.docx]

Otology

| **Society** | **Link ID** | **Title** | **URL** |  |  |  |  |  |
| --- | --- | --- | --- | --- | --- | --- | --- | --- |
| AAO-HNS | AAO - 01 | Autoimmune Inner Ear Disease | <https://www.enthealth.org/conditions/autoimmune-inner-ear-disease/> | | | | |  |
|  | AAO - 02 | BPPV | https://www.enthealth.org/conditions/benign-paroxysmal-positional-vertigo-bppv/ | | | | |  |
|  | AAO - 03 | Cholesteatoma | <https://www.enthealth.org/conditions/cholesteatoma/> | | | |  |  |
|  | AAO - 04 | Conductive Hearing Loss | <https://www.enthealth.org/conditions/conductive-hearing-loss/> | | | |  |  |
|  | AAO - 05 | Earaches | <https://www.enthealth.org/conditions/earaches/> | | |  |  |  |
|  | AAO - 06 | Ears + Altitude | <https://www.enthealth.org/conditions/ears-and-altitude-barotrauma/> | | | | |  |
|  | AAO - 07 | Earwax | <https://www.enthealth.org/conditions/earwax-cerumen-impaction/> | | | |  |  |
|  | AAO - 08 | Hyperacusis | https://www.enthealth.org/conditions/hyperacusis/ | | | |  |  |
|  | AAO - 09 | Labyrinthitis | https://www.enthealth.org/conditions/labyrinthitis/ | | | |  |  |
|  | AAO - 10 | Meniere's Disease | https://www.enthealth.org/conditions/menieres-disease/ | | | |  |  |
|  | AAO - 11 | Otosclerosis | <https://www.enthealth.org/conditions/otosclerosis/> | | | |  |  |
|  | AAO - 12 | Pediatric Hearing Loss | <https://www.enthealth.org/conditions/pediatric-hearing-loss/> | | | |  |  |
|  | AAO - 13 | Sensorineural Hearing Loss | <https://www.enthealth.org/conditions/sensorineural-hearing-loss/> | | | |  |  |
|  | AAO - 14 | Swimmer's Ears | <https://www.enthealth.org/conditions/swimmers-ear-otitis-externa/> | | | | |  |
|  | AAO - 15 | Tinnitus | <https://www.enthealth.org/conditions/tinnitus/> | | |  |  |  |
|  | AAO - 16 | Vestibular Schwannoma | <https://www.enthealth.org/conditions/vestibular-schwannoma-acoustic-neuroma/> | | | | |  |
|  |  |  |  |  |  |  |  |  |
| CSOHNS | CSO - 01 | Myringotomy and Tubes | <https://www.entcanada.org/education/general-public/public-information-sheets-2/ears/myringotomy-tubes/> | | | | | |
|  | CSO - 02 | Otology | <https://www.entcanada.org/education/general-public/public-information-sheets-2/ears/otology/> | | | | | |
|  | CSO - 03 | Dizziness | <https://www.entcanada.org/education/general-public/public-information-sheets-2/ears/dizziness-vertigo/> | | | | | |
|  |  |  |  |  |  |  |  |  |
| ENT UK | UK - 01 | Cholesteatoma/Mastoid Operations | <https://www.entuk.org/cholesteatoma-mastoid-operations> | | | |  |  |
|  | UK - 02 | Conditions and Surgery of the Ear | <https://www.entuk.org/conditions-and-surgery-ear> | | |  |  |  |
|  | UK - 03 | Dizziness | <https://www.entuk.org/dizziness> | |  |  |  |  |
|  | UK - 04 | Glue Ear | <https://www.entuk.org/glue-ear-ome> | | |  |  |  |
|  | UK - 05 | Grommets | <https://www.entuk.org/grommets> | | |  |  |  |
|  | UK - 06 | Hearing Aids and how to get one | <https://www.entuk.org/hearing-aids-and-how-get-one> | | | |  |  |
|  | UK - 07 | Hearing and Deafness | <https://www.entuk.org/hearing-and-deafness> | | |  |  |  |
|  | UK - 08 | Hearing Loss | <https://www.entuk.org/hearing-loss> | | |  |  |  |
|  | UK - 09 | Otitis Media | <https://www.entuk.org/middle-ear-infections> | | |  |  |  |
|  | UK - 10 | Otitis Externa | <https://www.entuk.org/outer-ear-infections> | | |  |  |  |
|  | UK - 11 | Mastoid Surgery | <https://www.entuk.org/mastoid-surgery> | | |  |  |  |
|  | UK - 12 | Meniere's Disease | <https://www.entuk.org/meni%c3%a8re%e2%80%99s-disease> | | | |  |  |
|  | UK - 13 | Otosclerosis and Stapedotomy | <https://www.entuk.org/otosclerosis-and-stapedotomy> | | | |  |  |
|  | UK - 14 | Perforated Eardrum | <https://www.entuk.org/perforated-eardrum-myringoplasty> | | | |  |  |
|  | UK - 15 | Tinnitus | <https://www.entuk.org/tinnitus-ringing-ears> | | |  |  |  |
|  | UK - 16 | Vertigo | <https://www.entuk.org/vertigo> | |  |  |  |  |
|  |  |  |  |  |  |  |  |  |
| AAO-HNS  Rhinology | AAO - 17 | Deviated Septum | <https://www.enthealth.org/conditions/deviated-septum/> | | | |  |  |
|  | AAO - 18 | Fungal Sinustitis | <https://www.enthealth.org/conditions/fungal-sinusitis/> | | | |  |  |
|  | AAO - 19 | Geriatric Rhinitis | <https://www.enthealth.org/conditions/geriatric-rhinitis/> | | | |  |  |
|  | AAO - 20 | Hyposmia and Anosmia | <https://www.enthealth.org/conditions/hyposmia-and-anosmia/> | | | |  |  |
|  | AAO - 21 | Nosebleeds | <https://www.enthealth.org/conditions/nosebleeds/> | | | |  |  |
|  | AAO - 22 | Pediatric Sinusitis | <https://www.enthealth.org/conditions/pediatric-sinusitis/> | | | |  |  |
|  | AAO - 23 | Rhinitis | <https://www.enthealth.org/conditions/rhinitis/> | | |  |  |  |
|  | AAO - 24 | Sinus Headaches | <https://www.enthealth.org/conditions/sinus-headaches/> | | | |  |  |
|  | AAO - 25 | Sinusitis | <https://www.enthealth.org/conditions/sinusitis/> | | |  |  |  |
|  | AAO - 26 | Turbinate Hypertrophy | <https://www.enthealth.org/conditions/turbinate-hypertrophy/> | | | |  |  |
|  |  |  |  |  |  |  |  |  |
| CSOHNS | CSO - 04 | Acute Sinusitis | <https://www.entcanada.org/education/general-public/public-information-sheets-2/nose/acute-sinusitis/> | | | | | |
|  | CSO - 05 | Chronic Sinusitis | <https://www.entcanada.org/education/general-public/public-information-sheets-2/nose/chronic-sinsitis/> | | | | | |
|  | CSO - 06 | Endoscopic Sinus Surgery | <https://www.entcanada.org/education/general-public/public-information-sheets-2/nose/endoscopic-sinus-surgery/> | | | | | |
|  | CSO - 07 | The nose in the winter | <https://www.entcanada.org/education/general-public/public-information-sheets-2/nose/nose-winter/> | | | | | |
|  |  |  |  |  |  |  |  |  |
| ENT UK | UK - 17 | Blocked Nose | <https://www.entuk.org/blocked-nose> | | |  |  |  |
|  | UK - 18 | Catarrh | <https://www.entuk.org/catarrh> | |  |  |  |  |
|  | UK - 19 | Flexible Nasal Endoscopy | <https://www.entuk.org/flexible-nasal-endoscopy> | | |  |  |  |
|  | UK - 20 | Functional Endoscopic Sinus Surgery | <https://www.entuk.org/functional-endoscopic-sinus-surgery-fess> | | | |  |  |
|  | UK - 21 | Nasal Polyps | <https://www.entuk.org/nasal-polyps> | | |  |  |  |
|  | UK - 22 | Epistaxis | <https://www.entuk.org/nosebleeds-epistaxis> | | |  |  |  |
|  | UK - 23 | Rhinosinusitis and Milk Allergy | <https://www.entuk.org/rhino-sinusitis-and-dairy-allergy> | | | |  |  |
|  | UK - 24 | Rhinosinusitis in children | <https://www.entuk.org/rhino-sinusitis-children> | | |  |  |  |
|  | UK - 25 | Sinus Infection | <https://www.entuk.org/sinus-infection-sinusitis> | | |  |  |  |
|  | UK - 26 | Septal Surgery | <https://www.entuk.org/surgery-node-septal-surgery> | | | |  |  |
|  | UK - 27 | Tumors of the Nose | <https://www.entuk.org/tumours-nose> | | |  |  |  |
| Head and Neck Oncology |  |  |  |  |  |  |  |  |
| AAO-HNS | AAO - 27 | Vocal Cord Paralysis | <https://www.enthealth.org/conditions/vocal-cord-fold-paralysis/> | | | |  |  |
|  | AAO - 28 | Hoarseness | <https://www.enthealth.org/conditions/hoarseness/> | | | |  |  |
|  | AAO - 29 | Dysphagia | <https://www.enthealth.org/conditions/dysphagia/> | | |  |  |  |
|  | AAO - 30 | Fine Needle Aspiration | <https://www.enthealth.org/conditions/fine-needle-aspiration/> | | | |  |  |
|  | AAO - 31 | Goiter | <https://www.enthealth.org/conditions/goiter/> | | |  |  |  |
|  | AAO - 32 | Grave's Disease | <https://www.enthealth.org/conditions/graves-disease/> | | | |  |  |
|  | AAO - 33 | Head and Neck Cancer | <https://www.enthealth.org/conditions/head-and-neck-cancer/> | | | |  |  |
|  | AAO - 34 | Human Papillomavirus | <https://www.enthealth.org/conditions/human-papillomavirus-hpv/> | | | |  |  |
|  | AAO - 35 | Hyperthyroidism | <https://www.enthealth.org/conditions/hyperthyroidism/> | | | |  |  |
|  | AAO - 36 | Neck Mass in Adults | <https://www.enthealth.org/conditions/evaluation-of-neck-mass-in-adults/> | | | | |  |
|  | AAO - 37 | Pediatric Thyroid Cancer | <https://www.enthealth.org/conditions/pediatric-thyroid-cancer/> | | | |  |  |
|  | AAO - 38 | Skin Cancer | <https://www.enthealth.org/conditions/skin-cancer/> | | | |  |  |
|  | AAO - 39 | Thyroid Cancer | <https://www.enthealth.org/conditions/thyroid-cancer/> | | | |  |  |
|  | AAO - 40 | Thyroid Nodules | <https://www.enthealth.org/conditions/thyroid-nodules/> | | | |  |  |
|  | AAO - 41 | Laryngeal Cancer | <https://www.enthealth.org/conditions/voice-box-laryngeal-cancer/> | | | |  |  |
|  |  |  |  |  |  |  |  |  |
| CSOHNS | CSO - 08 | Thyroid/Parathyroid Disease | <https://www.entcanada.org/education/general-public/public-information-sheets-2/head-neck/disorders-thyroidparathyroid-glands/> | | | | | |
|  | CSO - 09 | Head and Neck Cancer | <https://www.entcanada.org/education/general-public/public-information-sheets-2/head-neck/head-neck-cancer/> | | | | | |
|  | CSO - 10 | Masses and Cysts of the Neck | <https://www.entcanada.org/education/general-public/public-information-sheets-2/head-neck/masses-cysts-neck/> | | | | | |
|  |  |  |  |  |  |  |  |  |
| ENT UK | UK - 28 | Hoarseness | <https://www.entuk.org/hoarseness> | | |  |  |  |
|  | UK - 29 | Microlaryngoscopy | <https://www.entuk.org/microlaryngoscopy-and-oesophagoscopy> | | | | |  |
|  | UK - 30 | Facial Skin Lesions | <https://www.entuk.org/facial-skin-lesions> | | |  |  |  |
|  | UK - 31 | Head and Neck Cancer | <https://www.entuk.org/head-and-neck-cancer> | | |  |  |  |
|  | UK - 32 | Human Papillomavirus | <https://www.entuk.org/human-papilloma-virus-hpv> | | | |  |  |
|  | UK - 33 | Neck Dissection | <https://www.entuk.org/neck-dissection> | | |  |  |  |
|  | UK - 34 | Parotid Surgery | <https://www.entuk.org/parotid-surgery> | | |  |  |  |
|  | UK - 35 | Submandibular Gland Surgery | <https://www.entuk.org/submandibular-gland-surgery> | | | |  |  |
|  | UK - 36 | Thyroid Surgery | <https://www.entuk.org/thyroid-surgery> | | |  |  |  |
| General / Laryngology/ Pediatrics/ Facial Plastics |  |  |  |  |  |  |  |  |
| AAO-HNS | AAO - 42 | Ramsay-Hunt Syndrome | <https://www.enthealth.org/conditions/ramsay-hunt-syndrome/> | | | |  |  |
|  | AAO - 43 | Bell's Palsy | <https://www.enthealth.org/conditions/bells-palsy/> | | |  |  |  |
|  | AAO - 44 | Nasal Fractures | <https://www.enthealth.org/conditions/nasal-fractures/> | | | |  |  |
|  | AAO - 45 | Post-nasal drip | <https://www.enthealth.org/conditions/post-nasal-drip/> | | | |  |  |
|  | AAO - 46 | Aging and Swallowing | <https://www.enthealth.org/conditions/aging-and-swallowing/> | | | |  |  |
|  | AAO - 47 | Aspiration | <https://www.enthealth.org/conditions/aspiration/> | | |  |  |  |
|  | AAO - 48 | Dysgeusia | <https://www.enthealth.org/conditions/dysgeusia/> | | |  |  |  |
|  | AAO - 49 | Cricopharyngeal Muscle Dysfunction | <https://www.enthealth.org/conditions/cricopharyngeal-muscle-dysfunction/> | | | | |  |
|  | AAO - 50 | GERD and LPR | <https://www.enthealth.org/conditions/gerd-and-lpr/> | | | |  |  |
|  | AAO - 51 | Pediatric GERD | <https://www.enthealth.org/conditions/pediatric-gastroesophageal-reflux-disease-gerd/> | | | | | |
|  | AAO - 52 | Sore Throats | <https://www.enthealth.org/conditions/sore-throats/> | | | |  |  |
|  | AAO - 53 | Spasmodic Dysphonia | <https://www.enthealth.org/conditions/spasmodic-dysphonia/> | | | |  |  |
|  | AAO - 54 | Tonsillitis | <https://www.enthealth.org/conditions/tonsillitis/> | | |  |  |  |
|  | AAO - 55 | Zenker's Diverticulum | <https://www.enthealth.org/conditions/zenkers-diverticulum/> | | | |  |  |
|  | AAO - 56 | Snoring and Sleep Disorders | <https://www.enthealth.org/conditions/snoring-sleeping-disorders-and-sleep-apnea/> | | | | |  |
|  | AAO - 57 | Tonsils and Adenoids | <https://www.enthealth.org/conditions/tonsils-and-adenoids/> | | | |  |  |
|  | AAO - 58 | Cleft Palate/ Cleft Lip | <https://www.enthealth.org/conditions/cleft-palate/> | | | |  |  |
|  | AAO - 59 | Pediatric Sleep Disordered Breathing | <https://www.enthealth.org/conditions/pediatric-sleep-disordered-breathing/> | | | | |  |
|  | AAO - 60 | Salivary Gland Disorders | <https://www.enthealth.org/conditions/salivary-gland-disorders/> | | | |  |  |
|  | AAO - 61 | Sialadenitis | <https://www.enthealth.org/conditions/sialadenitis/> | | | |  |  |
|  | AAO - 62 | Asthma | <https://www.enthealth.org/conditions/asthma/> | | |  |  |  |
|  | AAO - 63 | Temporal-Mandibular Joint Pain | <https://www.enthealth.org/conditions/temporo-mandibular-joint-tmj-pain/> | | | | |  |
|  |  |  |  |  |  |  |  |  |
| CSOHNS | CSO - 11 | Cosmetic Ear Surgery | <https://www.entcanada.org/education/general-public/public-information-sheets-2/ears/cosmetic-surgery-ears/> | | | | | |
|  | CSO - 12 | Adenoidectomy | <https://www.entcanada.org/education/general-public/public-information-sheets-2/nose/adenoidectomy/> | | | | | |
|  | CSO - 13 | Allergies | <https://www.entcanada.org/education/general-public/public-information-sheets-2/nose/allergies/> | | | | | |
|  | CSO - 14 | Breathing Problems in Children | <https://www.entcanada.org/education/general-public/public-information-sheets-2/nose/breathing-problems-children/> | | | | | |
|  | CSO - 15 | Rhinoplasty | <https://www.entcanada.org/education/general-public/public-information-sheets-2/nose/rhinoplasty/> | | | | | |
|  | CSO - 16 | Disorders of the Salivary Glands | <https://www.entcanada.org/education/general-public/public-information-sheets-2/throat/disorders-salivary-glands/> | | | | | |
|  | CSO - 17 | Snoring | <https://www.entcanada.org/education/general-public/public-information-sheets-2/throat/snoring/> | | | | | |
|  | CSO - 18 | Tonsillectomy | <https://www.entcanada.org/education/general-public/public-information-sheets-2/throat/tonsillectomy/> | | | | | |
|  | CSO - 19 | Face and Neck Trauma | <https://www.entcanada.org/education/general-public/public-information-sheets-2/head-neck/face-neck-trauma/> | | | | | |
|  | CSO - 20 | Temporo-Mandibular Joint | <https://www.entcanada.org/education/general-public/public-information-sheets-2/head-neck/temporo-mandibular-joint-tmj/> | | | | | |
|  |  |  |  |  |  |  |  |  |
| ENT UK | UK - 37 | Protruding Ears | <https://www.entuk.org/protruding-ears-bat-ears-pinnaplasty> | | | |  |  |
|  | UK - 38 | Hayfever | <https://www.entuk.org/hayfever> | |  |  |  |  |
|  | UK - 39 | Nasal Injuries | <https://www.entuk.org/nasal-injuries> | | |  |  |  |
|  | UK - 40 | Rhinoplasty | <https://www.entuk.org/surgery-node-rhinoplasty> | | |  |  |  |
|  | UK - 41 | Adenoid Surgery | <https://www.entuk.org/adenoid-surgery> | | |  |  |  |
|  | UK - 42 | Adult Tonsil Surgery | <https://www.entuk.org/adult-tonsil-surgery> | | |  |  |  |
|  | UK - 43 | Children Tonsil Surgery | <https://www.entuk.org/children-tonsil-surgery> | | |  |  |  |
|  | UK - 44 | Snoring and OSA | <https://www.entuk.org/snoring-and-sleep-apnoea> | | |  |  |  |
|  | UK - 45 | Sore Throat | <https://www.entuk.org/sore-throat> | | |  |  |  |
