# Supplementary material for: Readability of the American, Canadian, and British Otolaryngology–Head and Neck Surgery Societies’ Patient Materials
Source: Otolaryngol Head Neck Surg. 2021 Aug 10;166(5):862–8. doi: 10.1177/01945998211033254 (PMC9066686; doi:10.1177/01945998211033254)
Supplement: sj-docx-2-oto-10.1177_01945998211033254 – Supplemental material for Readability of the American, Canadian, and British Otolaryngology–Head and Neck Surgery Societies’ Patient Materials [file sj-docx-2-oto-10.1177_01945998211033254.docx]

**Supplementary Material 2: Instruments and calculations used to assess readability**

| Assessment Scale | Formula |
| --- | --- |
| FRE | FRE = 206.835 − (84.6 × average # of syllables per word) − (1.015 × average # of words per sentence) |
| FKG | FKG = (11.8 × average # of syllables per word) + (0.39 ×  average # of words per sentence)− 15.59 |
| SMOG | SMOG  = 1.0430 Number of polysyllabic words 30number of sentences  + 3.1291 |

FRE: Flesch-Kincaid Reading Ease, FKG: Flesch Kincaid Grade Level, and SMOG: Simple Measure of Gobbledygook Index
