# Supplementary material for: Readability of the American, Canadian, and British Otolaryngology–Head and Neck Surgery Societies’ Patient Materials
Source: Otolaryngol Head Neck Surg. 2021 Aug 10;166(5):862–8. doi: 10.1177/01945998211033254 (PMC9066686; doi:10.1177/01945998211033254)
Supplement: sj-docx-3-oto-10.1177_01945998211033254 – Supplemental material for Readability of the American, Canadian, and British Otolaryngology–Head and Neck Surgery Societies’ Patient Materials [file sj-docx-3-oto-10.1177_01945998211033254.docx]

**Supplemental Material 3: Examples of current medical text found across AAO-HNS, CSOHNS and ENT UK and how they can be made more readable for readers.**

| Original Source | Original Text | FRE, FKGL, and SMOG scores of Original Text | Modified Text | FRE, FKGL and SMOG scores of Modified Text |
| --- | --- | --- | --- | --- |
| AAO-HNS  Link: <https://www.enthealth.org/conditions/vocal-cord-fold-paralysis/> | People have one set of two vocal cords, also known as vocal folds, that work together in your voice box to produce sound. They open when you breathe in to let the air flow through your lungs, and they close and vibrate when you speak (this is called phonation).To produce adequate voice, both vocal cords should move toward each other and close completely to vibrate together (this is called glottic closure). | FRE: 69.7  FKGL: 9.5  SMOG: 10.5 | People have two vocal cords. These are sometimes called vocal folds. These cords allow us to speak. During breathing, the vocal cords remain open. During speech, both vocal cords come together and vibrate. | FRE: 74.5  FKGL: 4.5  SMOG: 6.7 |
| CSOHNS  https://www.entcanada.org/learning/general-public/public-information-sheets-2/nose/endoscopic-sinus-surgery/ | Sinus surgery is indicated in certain individuals who suffer from chronic sinusitis and in a small number of people who suffer from acute sinusitis. Surgery is usually the last resort after medical treatment has failed to improve the patient’s symptoms. | FRE: 25.8  FKGL: 14.6  SMOG: 14.6 | Patients who undergo sinus surgery have long-term sinus symptoms. They can also have many sinus infections. Surgery is not recommended until other treatments have been tried. These can include steroids. Steroids come as either a spray in the nose or a pill. If there is no improvement with these, surgery is considered. | FRE=61.4,  FKGL=6.9, SMOG=9.7 |
| ENT-UK  https://www.entuk.org/nasal-polyps | Nasal polyps blocking the nose can be removed surgically and this often helps the patient to breathe better. In three out of four patients the polyps come back after an average period of four years.  If they return repeatedly the sinuses can be cleaned out and opened up and it is thought that this gives a longer period before they return. Local medical treatment is often still needed using anti-inflammatory sprays or drops | FRE: 56.6  FKGL: 10.0  SMOG: 11.2 | Nasal polyps can be removed through surgery. After surgery, patients can often breathe better. In three out of four patients, the polyps can grow back. This usually takes about four years. If they continue to grow back, more extensive sinus surgery can be performed. While this often helps, nasal sprays and drops are still needed after surgery. | FRE: 66.6  FKGL: 6.3  SMOG: 9.3 |

*AAO-HNS: American Academy of Otolaryngology-Head and Neck Surgery, CSOHNS: Canadian Society of Otolaryngology-Head and Neck Surgery, ENT UK: Ear Nose and Throat United Kingdom.*
